# Two-dimensional echocardiography after return of spontaneous circulation and its association with in-hospital survival after in-hospital cardiopulmonary resuscitation

**DOI:** 10.1038/s41598-019-56153-z

**Published:** 2020-01-08

**Authors:** In-Ae Song, Jun Kwon Cha, Tak Kyu Oh, You Hwan Jo, Yeonyee E. Yoon

**Affiliations:** 1 Department of Anesthesiology and Pain Medicine, Seoul National University Bundang Hospital, Seongnam, South Korea; 2 Department of Emergency Medicine, Hallym University Sacred Heart Hospital, Chuncheon, South Korea; 3 Department of Emergency Medicine, Seoul National University Bundang Hospital, Seongnam, South Korea; 4 Department of Cardiology, Cardiovascular Centre, Seoul National University Bundang Hospital, Seongnam, South Korea

**Keywords:** Interventional cardiology, Quality of life, Risk factors

## Abstract

This retrospective cohort study investigated the association between in-hospital survival and two-dimensional (2D) echocardiography within 24 hours after the return of spontaneous circulation (ROSC) in patients who underwent in-hospital cardiopulmonary resuscitation (ICPR) after in-hospital cardiopulmonary arrest (IHCA). The 2D-echo and non-2D-echo groups comprised eligible patients who underwent transthoracic 2D echocardiography performed by the cardiology team within 24 hours after ROSC and those who did not, respectively. After propensity score (PS) matching, 142 and 284 patients in the 2D-echo and non-2D-echo groups, respectively, were included. A logistic regression analysis showed that the likelihood of in-hospital survival was 2.35-fold higher in the 2D-echo group than in the non-2D-echo group (*P* < 0.001). Regarding IHCA aetiology, in-hospital survival after cardiac arrest of a cardiac cause was 2.51-fold more likely in the 2D-echo group than in the non-2D-echo group (*P* < 0.001), with no significant inter-group difference in survival after cardiac arrest of a non-cardiac cause (*P* = 0.120). In this study, 2D echocardiography performed within 24 hours after ROSC was associated with better in-hospital survival outcomes for patients who underwent ICPR for IHCA with a cardiac aetiology. Thus, 2D echocardiography may be performed within 24 hours after ROSC in patients experiencing IHCA to enable better treatment.

## Introduction

In-hospital cardiac arrest (IHCA) is an important determinant of in-hospital mortality^1^. According to recent reports, 290,000 cases of IHCA occur annually in the U.S^2^. Among these, approximately 2.73 patients per 1000 patients with IHCA who undergo in-hospital cardiopulmonary resuscitation (ICPR) are elderly inpatients^3,4^. Despite steady increases in the post-IHCA survival rate due to improvements in the quality of patient care^5^, survival remains a significant challenge in this population^2^.

Two-dimensional (2D) echocardiography is a rapid, safe, and non-invasive modality used to examine the motion of the heart. In addition to its usefulness for treating and evaluating patients in intensive care unit (ICU) settings^6^, 2D echocardiography has been reported to play a major role in vasopressor dose adjustments or fluid management during the treatment of patients with shock^7,8^. In patients with IHCA, 2D echocardiography has been shown to facilitate the rapid detection or exclusion of various aetiologies during the peri-resuscitative period and to help physicians identify targets for possible correction^9^. Furthermore, 2D echocardiography is useful for the diagnosis and treatment of post-arrest myocardial dysfunction (PAMD), a common condition affecting patients who achieve return of spontaneous circulation (ROSC) after IHCA^10–12^. Accordingly, 2D echocardiography may be useful for improving post-ROSC survival for patients who undergo ICPR for IHCA. However, available information related to this topic remains insufficient.

Therefore, this study aimed to investigate the association between in-hospital survival and the performance of 2D echocardiography within 24 hours after ROSC in patients who underwent ICPR after IHCA. Using propensity score (PS) matching, we compared patients who did and did not undergo 2D echocardiography. We hypothesised that those who underwent 2D echocardiography would have better in-hospital survival rates.

## Results

### Characteristics of study subjects

**Figure 1:**
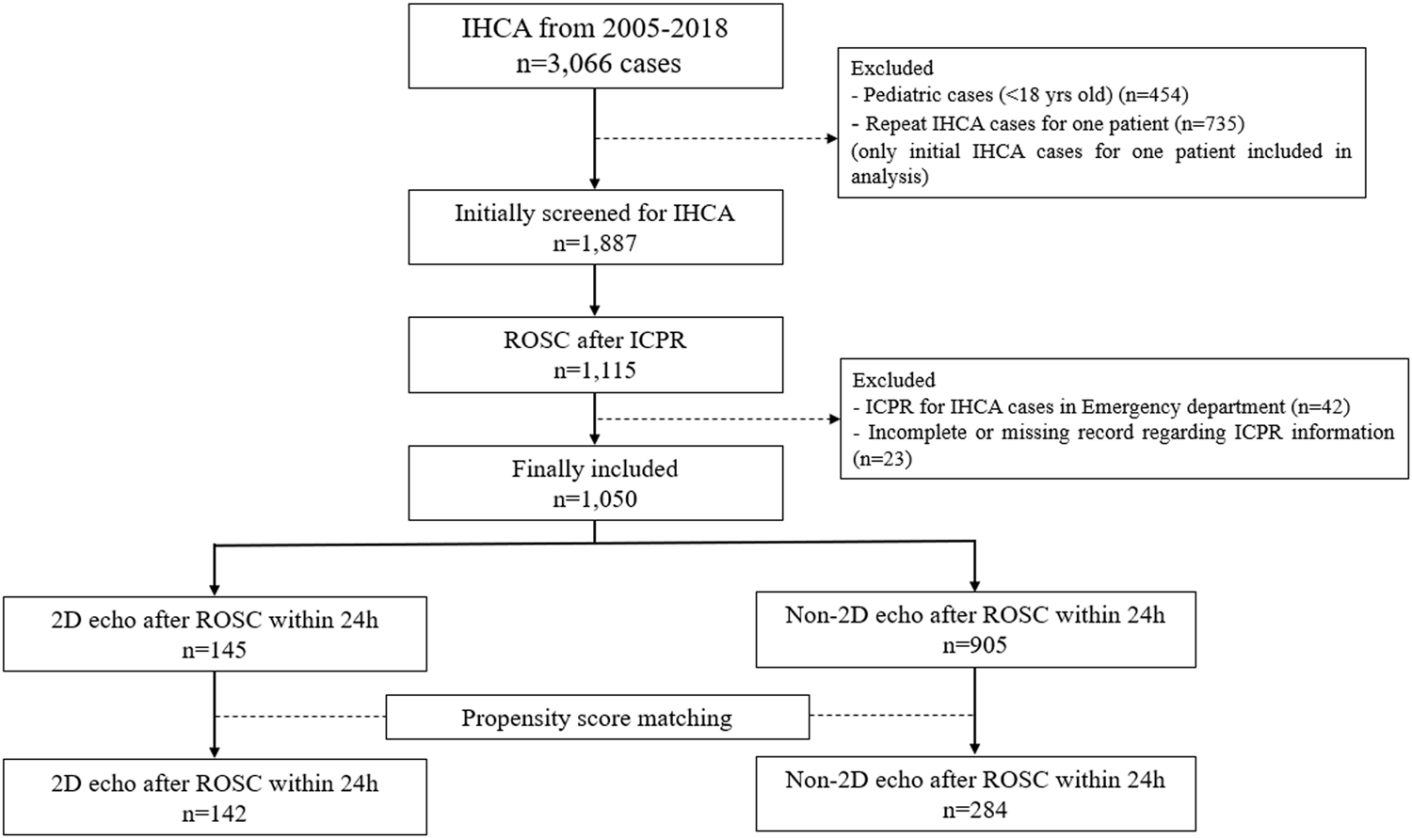
Flow chart of patient selection. IHCA, in-hospital cardiac arrest; ICPR, in-hospital cardiopulmonary resuscitation; 2D echo, two-dimensional echocardiography; ROSC, return of spontaneous circulation.

A total of 3,066 cases of IHCA were recorded at the study institution from January 2005 to December 2018. Of these, 454 cases involving paediatric patients and 735 cases involving repeated events of IHCA for a single patient during the study period were excluded. Accordingly, 1,887 patients with a first IHCA event were initially screened for study inclusion. Of those, 1,115 achieved ROSC after ICPR. After excluding 42 patients who underwent ICPR in the emergency department and 23 patients whose ICPR information was incomplete or missing, 1,050 patients were stratified according to their 2D echocardiography status. Eligible patients who underwent 2D echocardiography were assigned to the 2D-echo group, and those who did not undergo 2D echocardiography were assigned to the non-2D-echo group. The initial 2D-echo and non-2D-echo groups included 145 (13.8%) and 905 patients (86.2%), respectively. After PS matching, 142 and 284 patients remained in the 2D-echo and non-2D-echo groups, respectively (Fig. 1). The baseline characteristics of patients included in the analyses before and after PS matching are shown in Table 1. After PS matching, all covariates were well-balanced between the 2D-echo and non-2D-echo groups, with an absolute value of the standardised mean difference (ASD) of <0.1. The PS distributions of the two groups were also similar, as demonstrated by a comparison between pre- and post-PS values (Fig. S1).

**Table 1:** Comparison of characteristics between patients who underwent echocardiography within 24 hours after ROSC from ICPR.

Variable	Entire cohort (n = 1,050)			PS-matched cohort (n = 426)		
	2D-echo group n = 145	Non-2D echo group n = 905	ASD	2D-echo group n = 142	Non-2D echo group n = 284	ASD
Age, yr	67.5 (15.2)	66.7 (14.5)	0.056	67.4 (15.3)	67.3 (13.4)	0.011
Sex, male	88 (60.7)	589 (65.1)	0.089	87 (61.3)	167 (58.8)	0.050
BMI^a^ before multiple imputation (missing n:146)	23.0 (4.5)	21.9 (4.5)				
BMI^a^ after multiple imputation	23.0 (4.4)	21.9 (4.3)	0.260	22.9 (4.3)	22.7 (4.3)	0.061
Charlson comorbidity index at IHCA	1.0 (1.2)	0.9 (1.2)	0.060	1.0 (1.2)	0.9 (1.3)	0.064
Admitting department at IHCA			0.029			0.047
Internal medicine	82 (56.6)	529 (58.5)		82 (57.7)	167 (58.8)	
Surgical department	24 (16.6)	140 (15.5)		23 (16.2)	41 (14.4)	
Others	39 (26.9)	236 (26.1)		37 (26.1)	76 (26.8)	
Year of IHCA			0.533			0.073
2006–2010	26 (17.9)	350 (38.7)		26 (18.3)	60 (21.1)	
2011–2014	73 (50.3)	313 (34.6)		71 (50.0)	139 (48.9)	
2015–2018	46 (31.7)	242 (26.7)		45 (31.7)	85 (29.9)	
Time of IHCA			0.006			0.032
Day time (07:00–22:59)	106 (73.1)	664 (73.4)		103 (72.5)	210 (73.9)	
Night time (23:00–06:59)	39 (26.9)	241 (26.6)		39 (27.5)	74 (26.1)	
Weekday of IHCA			0.513			0.053
Monday-Friday	127 (87.6)	639 (70.6)		124 (87.3)	253 (89.1)	
Saturday, Sunday, and legal holiday	18 (12.4)	266 (29.4)		18 (12.7)	31 (10.9)	
First detector of IHCA			0.119			0.025
Medical staff	132 (91.0)	793 (87.6)		129 (90.8)	260 (91.5)	
Non-medical staff	13 (9.0)	112 (12.4)		13 (9.2)	24 (8.5)	
Location of IHCA			0.029			0.061
General ward	93 (64.1)	543 (60.0)		92 (64.8)	182 (64.1)	
Intensive care unit	23 (15.9)	114 (12.6)		23 (16.2)	41 (14.4)	
Other places in hospital	29 (20.0)	248 (27.4)		27 (19.0)	61 (21.5)	
Time from arrest to ICPR			0.228			0.087
<1 min	106 (73.1)	631 (69.7)		105 (73.9)	203 (71.5)	
1–2 min	17 (11.7)	95 (10.5)		16 (11.3)	40 (14.1)	
2–3 min	11 (7.6)	47 (5.2)		10 (7.0)	21 (7.4)	
3–4 min	4 (2.8)	37 (4.1)		4 (2.8)	8 (2.8)	
4–5 min	0 (0.0)	7 (0.8)		0 (0.0)	0 (0.0)	
>5 min	7 (4.8)	88 (9.7)		7 (4.9)	12 (4.2)	
Duration of ICPR			0.209			0.069
0–5 min	46 (31.7)	266 (29.4)		46 (32.4)	91 (32.0)	
5–10 min	38 (26.2)	196 (21.7)		36 (25.4)	67 (23.6)	
10–15 min	26 (17.9)	143 (15.8)		26 (18.3)	57 (20.1)	
15–20 min	15 (10.3)	90 (9.9)		14 (9.9)	22 (7.7)	
20–30 min	9 (6.2)	91 (10.1)		9 (6.3)	20 (7.0)	
>30 min	11 (7.6)	119 (13.1)		11 (7.7)	27 (9.5)	
Placement of artificial airway during ICPR	64 (44.1)	305 (33.7)	0.209	62 (43.7)	120 (42.3)	0.028
Defibrillation during ICPR	42 (29.0)	246 (27.2)	0.039	41 (28.9)	80 (28.2)	0.016
Epinephrine injection during ICPR	94 (64.8)	422 (46.6)	0.380	92 (64.8)	183 (64.4)	0.007
Etiology of IHCA			0.369			0.027
Cardiac arrest	116 (80.0)	572 (63.2)		113 (79.6)	223 (78.5)	
Respiratory arrest	28 (19.3)	307 (33.9)		28 (19.7)	59 (20.8)	
Unknown	1 (0.7)	26 (2.9)		1 (0.7)	2 (0.7)	

Presented as number (percentage) or mean value (standard deviation).

^a^Because some patients have missing BMI data, we performed multiple imputation using PROC MI. After performing multiple imputations, 10 data sets are created, and each missing value is replaced by the average of BMI values replaced by 10 times.

ROSC, return of spontaneous circulation; PS, propensity score; ASD, absolute value of standardised mean difference; BMI, body mass index; IHCA, in-hospital cardiac arrest; ICPR, in-hospital cardiopulmonary resuscitation.

### Main results

**Figure 2:**
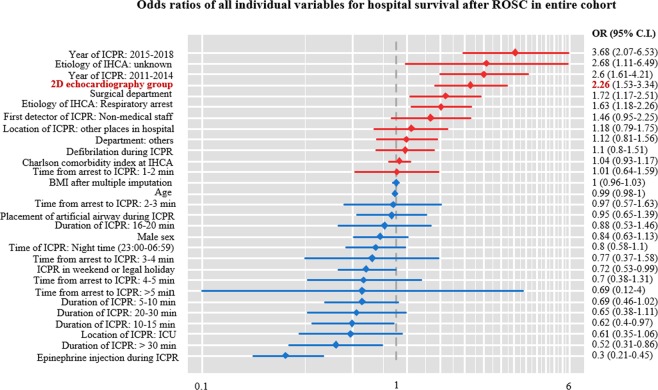
Multivariable logistic regression analysis of in-hospital survival after the return of spontaneous circulation (ROSC) for the entire cohort. ICPR, in-hospital cardiopulmonary resuscitation; IHCA, in-hospital cardiac arrest.

Table 2 presents the results of a comparative analysis for in-hospital survival between the 2D-echo and non-2D-echo groups before and after PS matching. In the PS-matched cohort, 49.3% (70/142) of patients in the 2D-echo group survived to discharge from the hospital, compared to only 29.2% (83/284) of those in the non-2D-echo group. In a logistic regression analysis of the PS-matched cohort, the in-hospital survival rate in patients in the 2D-echo group was 2.35-fold higher than that in patients in the non-2D-echo group (odds ratio [OR]: 2.35, 95% confidence interval [CI]: 1.55–3.57; *P* < 0.001). A multivariable logistic regression analysis of the entire cohort similarly revealed that the in-hospital survival rate in patients in the 2D-echo group was 2.26-fold higher than that in patients in the non-2D-echo group (OR: 2.26, 95% CI: 1.53–3.34; *P* < 0.001; Fig. 2). Table 3 presents the differences in in-hospital survival between the PS-matched 2D-echo and non-2D-echo groups according to the aetiology of IHCA. When the aetiology of IHCA was cardiac arrest of a cardiac cause (n = 336), the in-hospital survival rate in patients in the 2D-echo group was 2.51-fold higher than that in patients in the non-2D echo group (OR: 2.51, 95% CI: 1.56–4.04; P < 0.001). By contrast, no significant inter-group difference was observed in the comparison of cases of cardiac arrest of a non-cardiac cause (*P* = 0.120).

**Table 2:** In-hospital survival after ROSC before and after propensity score matching.

Variables	Survival rate		OR (95% CI)	*P*-value
**Unadjusted (entire cohort)**				
Non-2D echo group (n = 905)		308 / 905 (34.0%)	1	
2D echo group (n = 145)		73 / 145 (50.3%)	1.97 (1.38, 2.80)	<0.001
**Adjusted (PS-matched cohort)**				
Non-2D echo group (n = 284)		83 / 284 (29.2%)	1	
2D echo group (n = 142)		70 / 142 (49.3%)	2.35 (1.55, 3.57)	<0.001

ROSC, return of spontaneous circulation; OR, odds ratio; CI, confidence interval.

**Table 3:** In-hospital survival rate according to the aetiology of IHCA in propensity score-matched cohort.

Variables	Survival rate	OR (95% CI)	*P*-value
**Cardiac arrest of cardiac cause (n = 336)**			
Non-2D echo group (n = 223)	58/223 (26.0%)	1	
2D echo group (n = 113)	53/113 (46.9%)	2.51 (1.56, 4.04)	<0.001
**Cardiac arrest of non-cardiac cause (n = 90)**			
Non-2D echo group (n = 61)	25/61 (41.0%)	1	
2D echo group (n = 29)	17/29 (58.6%)	2.04 (0.83, 5.01)	0.120

IHCA, in-hospital cardiac arrest; OR, odds ratio; CI, confidence interval.

## Discussion

In this study, performing 2D echocardiography within 24 hours after ROSC was associated with a greater likelihood of survival at hospital discharge in a cohort of subjects who underwent ICPR due to IHCA. However, a subgroup analysis revealed that this association was only evident if the IHCA had a cardiac aetiology. This finding, which was demonstrated using both PS matching and multivariable-adjusted analyses of the entire cohort, is significant, because it suggests that 2D echocardiography should be performed within 24 hours of ROSC after IHCA to allow physicians to make more appropriate treatment decisions.

Our findings raise several points. First, and most importantly, PAMD was unlikely to be detected in patients who did not undergo 2D echocardiography within 24 hours after ROSC^10–12^. As rapid 2D echocardiography is required for the diagnosis of possible PAMD during the post-arrest period^13^, patients in our study who did not undergo 2D echocardiography within 24 hours were less likely to receive appropriate treatment for this condition. Second, 2D echocardiography can facilitate the rapid diagnosis or elimination of reversible causes of IHCA^9^, such as acute cardiac tamponade^14^, myocardial infarction^15^, or pulmonary embolism^16^. Therefore, such causes may not have been identified in patients who did not undergo 2D echocardiography (i.e., those in the non-2D echo group), whereas patients in the 2D-echo group were more likely to benefit from rapid detection and treatment. Third, 2D echocardiography may have helped to improve the quality of post-arrest care in patients with IHCA due to non-cardiac causes (e.g., sepsis or hypovolemia). Consistent with this possibility, 2D echocardiography has been reported to facilitate the management of septic and hypovolemic shock^7,8,17^.

Our findings directly contrast those of Jentzer *et al*., who reported that 2D echocardiographic findings (e.g., left ventricular ejection fraction) obtained within 24 hours after ROSC were not useful predictors of survival^18^. We note that these studies differed decisively with respect to patient selection. Our study included every patient who achieved ROSC after ICPR for IHCA, regardless of their 2D echocardiography status within 24 hours, and differences in survival to discharge were compared between the 2D-echo and non-2D-echo groups. In contrast, the study by Jentzer and colleagues only included patients who underwent 2D echocardiography and only investigated the associations between echocardiographic findings and survival. Our reinterpretation of their findings suggests that the 55 patients prospectively included in the study by Jentzer *et al*. were likely to have received reasonable treatment after ROSC in accordance with their 2D echocardiographic findings. Therefore, the general effects of appropriate treatment were unlikely to lead to significant differences in survival rates determined according to 2D echocardiographic findings in the study by Jentzer and colleagues.

We additionally performed a sensitivity analysis of our cohort according to the aetiology of IHCA. Interestingly, patients with cardiac IHCA of a non-cardiac cause (i.e., respiratory arrest) did not realise survival benefits from 2D echocardiography performed within 24 hours after ROSC. Pulmonary function and ventilator support are the conventional determinants of in-hospital survival in patients with respiratory failure^19^, and the benefits of 2D echocardiography in patients with acute respiratory distress syndrome have not been fully established^20^. In this context, our findings suggest that patients who underwent 2D echocardiography within 24 hours after respiratory arrest either were significantly less likely to change therapy or were more likely to receive a worse prognosis than were patients with cardiac arrest of a cardiac cause. Additional studies are needed to determine whether 2D echocardiography is useful for patients with respiratory arrest, given the associated time and financial costs.

We set 24 hours as the cut-off duration for 2D echocardiography to be performed after ROSC from ICPR to be defined as a ‘rapid test’. There are no clear guidelines outlining the most appropriate period for performing 2D echocardiography after ROSC from ICPR. However, a prospective study by Jentzer *et al*. evaluated 55 adults undergoing 2D echocardiography within 24 hours after ROSC from ICPR^18^, which is in line with our study. It is possible that performing 2D echocardiography much less than 24 hours after ROSC from ICPR might result in greater benefits for in-hospital survival than simply performing it sometime within 24 hours. Additional studies are needed to determine if that is true.

This study had some notable limitations. First, the study was retrospective, and the results may have been affected by various confounders. In our analysis, we used PS matching and multivariable adjustments to reduce these confounding effects. Second, this study was based on data from a single centre, and therefore the generalizability of the results may be limited. Third, although many variables were included as confounders in the PS matching and multivariable adjustments, additional unmeasured confounders may have affected outcomes. Lastly, most patients did not receive 2D echocardiography within 24 hours after ROSC due to a lack of medical resources in our hospital, so selection bias may have occurred. Therefore, this is a significant limitation of our study.

In conclusion, our findings showed that 2D echocardiography performed within 24 hours after ROSC was associated with better in-hospital survival outcomes in patients who underwent in-hospital CPR for IHCA. Moreover, this association remained significant only when the aetiology of IHCA was a cardiac cause. Our study suggests that 2D echocardiography should be performed within 24 hours after ROSC from IHCA to help ensure that patients receive the appropriate treatment.

## Methods

### Study design and setting

This retrospective cohort study was conducted in a single tertiary academic hospital. The study protocol was approved by the Institutional Review Board (IRB) of Seoul National University Bundang Hospital (SNUBH) (IRB approval number: B-1904/535-101). The IRB granted a waiver of the requirement to obtain informed consent, given the retrospective study design and the use of medical records from patients whose medical care has been completed. SNUBH is a tertiary academic hospital of the medical college of Seoul National University. It has 1,350 inpatient beds and a large teaching centre. As reported previously^21^, the average number of ICPR performed for IHCA cases per 1000 patients per month was 4.22 (SD: 1.13) from 2007 to 2016. The initial ROSC rate following ICPR from IHCA was reported to be 72.3% from 2012 to 2016^22^. A rapid response system has been in place since 2013 to reduce the incidence of IHCA at SNUBH^23^, and ICPR has been performed for IHCA by both the rapid response system and an on-duty resident team^24^.

### Data registry and study population

This study used data from electronic health records stored in the Bundang Hospital Electronic System for Total Care (BESTCare), the electronic medical record system of the SNUBH^25^. Data were extracted specifically from the CPR-data registry, which includes all cases of ICPR since 2005. The present study included all patients admitted to the SNUBH from 2005 to 2018 who developed IHCA, underwent ICPR, and achieved ROSC. Paediatric cases (<18 years) were excluded. If multiple IHCA events were recorded for a single patient during the indicated study period, only the first event was included in the analysis.

### Main independent variable: 2D echocardiography after ROSC

During the study period, the decision to perform 2D echocardiography after ROSC was made at the discretion of the attending or on-call physician and was not subject to a particular protocol of the SNUBH. Eligible patients who underwent transthoracic 2D echocardiography performed by a cardiology team within 24 hours after ROSC were defined as the 2D-echo group. All other eligible patients were defined as the non-2D-echo group. In our institution, not all patients underwent 2D echocardiography within 24 hours after ROSC from IHCA, due to a lack of resources, such as medical staff members and 2D echocardiography equipment.

### Dependent variable: in-hospital survival after ROSC

In this study, in-hospital survival was defined as survival to discharge from the hospital. This criterion was used to stratify all patients who achieved ROSC after ICPR.

### Measurements (potential confounders)

We collected the following data as potential confounders: physical characteristics (age, sex, body mass index [BMI]), Charlson comorbidity index at the time of IHCA, admitting department at the time of IHCA, year of ICPR, time of IHCA (day [07:00–22:59 h] or night [23:00–06:59 h]), weekday of IHCA, first detector of IHCA (medical staff or other), location of IHCA (general ward, ICU, or other hospital location), time from cardiac arrest to ICPR, duration of ICPR, placement of an artificial airway during ICPR, use of defibrillation during ICPR, use of epinephrine during ICPR, and the aetiology of IHCA (cardiac, respiratory, or unknown).

### Outcomes

This study aimed primarily to determine whether the in-hospital survival of patients who achieved ROSC after ICPR for IHCA would differ with respect to their 2D echocardiography status. The study also aimed to determine whether this outcome would differ according to the aetiology of IHCA.

### Statistical analysis

The baseline characteristics of patients included in the study are presented as means with standard deviations or numbers with percentages. Among all covariates, the only category with missing data was the BMI at IHCA (13.9%, 146/1,050). To reduce bias due to missing data^26^, we performed five multiple imputations to generate five datasets, and each missing value in the original dataset was replaced by the average of the five BMI values in each of the five datasets prior to performing PS matching. These five datasets were not included in the subsequent PS model and were not used to evaluate the consistency of PS matching.

Next, we performed PS matching, an effective method for reducing the effects of confounders in an observational study^27^. All covariates were matched at a 1:2 ratio using a calliper of 0.2 via the nearest-neighbour method, without replacement. A logistic regression analysis was generated to calculate the PSs as a logistic model, and the ASD was used to evaluate the balance between the 2D-echo and non-2D-echo groups before and after PS matching. The ASD was set to <0.1 to balance the two groups after PS matching.

After confirming that the covariates were well-balanced between the two groups in the PS-matched cohort, we performed a logistic regression analysis for in-hospital survival after ROSC, as well as two sensitivity analyses. In the first sensitivity analysis, we performed a multivariable logistic regression analysis of in-hospital survival after ROSC for the entire cohort to demonstrate the generalizability of the results from our PS-matched cohort to the entire hospital cohort and to explain the association of 2D echocardiography with in-hospital survival after ROSC, along with other important covariates rather than isolation. This analysis included all covariates, and the absence of multi-collinearity in the multivariable model was confirmed by variance inflation factors with values < 2.0. Hosmer–Lemeshow statistics were used to confirm the appropriate goodness of fit (*P* > 0.5). In the second sensitivity analysis, we investigated whether the association of in-hospital survival with 2D echocardiography status after ROSC would depend on the aetiology of IHCA.

The results of all logistic regression analyses are presented as ORs with 95% CIs. The results of a multivariable logistic regression analysis for in-hospital survival after ROSC in the entire cohort are presented as a Forest plot. All analyses were performed using R version 3.5.2 (R Project for Statistical Computing), and a *P* value < 0.05 was considered statistically significant.

### Ethical statement

This retrospective cohort study was conducted in a single tertiary academic hospital. The study protocol was approved by the Institutional Review Board (IRB) of Seoul National University Bundang Hospital (SNUBH) (IRB approval number: B-1904/535-101). This IRB granted a waiver of the requirement for informed consent, given the retrospective study design and the use of medical records from patients whose medical care has been completed.

## Supplementary information

Supplementary Information 

## Data Availability

The datasets used and/or analysed during the current study are available from the corresponding author upon reasonable request.
