# Supplementary material for: Two-dimensional echocardiography after return of spontaneous circulation and its association with in-hospital survival after in-hospital cardiopulmonary resuscitation
Source: Sci Rep. 2020 Jan 8;10:11. doi: 10.1038/s41598-019-56153-z (PMC6949305; doi:10.1038/s41598-019-56153-z)

Two-dimensional echocardiography after return of spontaneous circulation and its association with in-hospital survival after in-hospital cardiopulmonary resuscitation

Short running title: 2D ECG after ROSC and survival

In-Ae Song, M.D., Ph.D.[1§]; Jun Kwon Cha, M.D.[2§]; Tak Kyu Oh, M.D.[1*]; You Hwan Jo, M.D., Ph.D.[3]; and Yeonyee E. Yoon, M.D.[4]

[1]Department of Anesthesiology and Pain Medicine, Seoul National University Bundang Hospital

[2]Department of Emergency Medicine, Hallym University Sacred Heart Hospital

[3]Department of Emergency Medicine, Seoul National University Bundang Hospital

[4]Department of Cardiology, Cardiovascular Centre, Seoul National University Bundang Hospital

[§]equal contribution as co-first authors

***Corresponding author:** Tak Kyu Oh, MD

Department of Anaesthesiology and Pain Medicine, Seoul National University Bundang Hospital, Gumi-ro 173 Beon-gil, Bundang-gu, Seongnam 13620, Korea

Tel: +82-31-787-7499, Fax: +82-31-787-4063

Email: airohtak@hotmail.com

Supplementary Figure Caption

Fig. S1 Distribution of propensity scores before and after propensity score matching

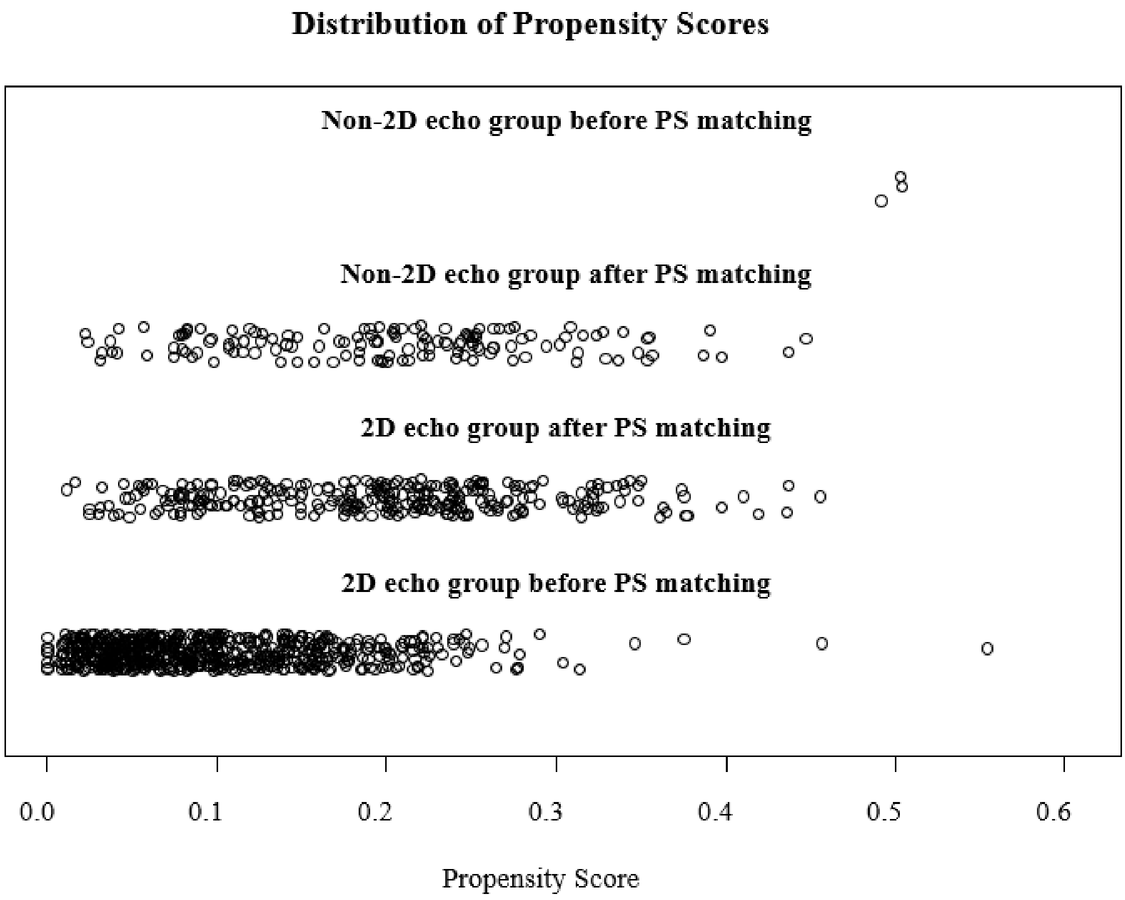

Supplement: Supplementary file 1 — Supplementary Information [file 41598_2019_56153_MOESM1_ESM.pdf]
